# Overexpression of bone morphogenetic protein 7 reduces oligodendrocytes loss and promotes functional recovery after spinal cord injury

**DOI:** 10.1111/jcmm.16832

**Published:** 2021-08-13

**Authors:** Shuxin Liu, Wei Zhang, Lin Yang, Fan Zhou, Peng Liu, Yaping Wang

**Affiliations:** ^1^ Department of Pain Management and Anesthesiology The Second Xiangya Hospital Central South University Changsha China; ^2^ Department of Disease Prevention and Control People's Liberation Army Joint Logistic Support Force 921th Hospital Changsha China

**Keywords:** BMP7, demyelination, oligodendrocytes, spinal cord injury

## Abstract

Spinal cord injury (SCI), as a severe disease with no effective therapeutic measures, has always been a hot topic for scientists. Bone morphogenetic protein 7 (BMP7), as a multifunctional cytokine, has been reported to exert protective effects on the nervous system. The present study aimed to investigate the neuroprotective effect and the potential mechanisms of BMP7 on rats that suffered SCI. Rat models of SCI were established by the modified Allen^'^s method. Adeno‐associated virus (AAV) was injected at T9 immediately before SCI to overexpress BMP7. Results showed that the expression of BMP7 decreased in the injured spinal cords that were at the same time demyelinated. AAV‐BMP7 partly reversed oligodendrocyte (OL) loss, and it was beneficial to maintain the normal structure of myelin. The intervention group showed an increase in the number of axons and Basso‐Beattie‐Bresnahan scores. Moreover, double‐labelled immunofluorescence images indicated p‐Smad1/5/9 and p‐STAT3 in OLs induced by BMP7 might be involved in the protective effects of BMP7. These findings suggest that BMP7 may be a feasible therapy for SCI to reduce demyelination and promote functional recovery.

## 1 INTRODUCTION

Spinal cord injury (SCI) is a severe disease with tremendous tissue degeneration and cell apoptosis that can result in relatively high morbidity and disability.^1^ Oligodendrocytes (OLs) are susceptible to apoptosis during both the acute phase^2^, ^3^ and the chronic phase of SCI.^4^, ^5^ OL death causes the demyelination of axons and eventually results in lasting neurological disability.^5^, ^6^, ^7^, ^8^ Therefore, protecting OLs and myelin may be a potential strategy for tissue repair and neurological recovery after SCI. How to protect OLs and myelin after SCI has already aroused the interest of many scientists.^9^

Bone morphogenetic protein 7 (BMP7) has been reported to reduce neuron loss,^10^, ^11^ induce axonal regeneration^12^, ^13^ and reduce brain infarct size^14^, ^15^, ^16^ in different models of neurological disease. In the mixed neuronal cultures, overexpression of BMP7 has profoundly increased the number of OLs.^17^ Researchers have proved that agmatine plays a key role in inducing oligodendrogenesis of rats that suffered SCI, which is likely associated with the upregulation of BMP7 in OLs.^18^ Moreover, a previous study of our team has validated the protective effects of BMP7 against OLs apoptosis induced by TNF‐α.^19^ In vivo, local overexpression of BMP7 has been reported to protect Schwann cells and can reduce demyelination induced by sciatic nerve injury as well as promote functional recovery.^20^ Based on the studies above, we speculate that BMP7 plays a role in protecting OLs after SCI.

In the current study, we examined the neuroprotection of BMP7 on SCI rats. Specifically, we focused on the influence of overexpressed BMP7 on the damage to OLs and myelin within the epicentre of injured spinal cords. Furthermore, the possible mechanisms of action of BMP7 were investigated.

## 2 MATERIALS AND METHODS

### 2.1 Animals and groups

Male Sprague Dawley rats (weighing 200–250 g), obtained from Central South University Animal Services, were housed (six rats per cage) with food and water, in a room with a 12:12‐h light:dark cycle and constant temperature and humidity. All of the experiments were conducted with the approval of the animal ethics committee of Xiangya Medical College of Central South University and in strict accordance with the National Institutes of Health Guide for the Care and Use of Laboratory Animals. In the first part of the study, 48 rats were divided into six groups randomly to explore the expression of proteins at indicated time points (*n* = 8). To investigate the transfection duration of AAV9, nine rats were divided into three groups: 1, 2 and 4 weeks (*n* = 3). To figure out the effects of AAV‐BMP7 on OLs, 48 rats were divided into four groups: sham, SCI, SCI + AAV‐GFP and SCI + AAV‐BMP7 (*n* = 12).

### 2.2 Construction and infusion of adeno‐associated virus

We chose AAV9, which is susceptible to transfect nerve system, to overexpress BMP7. AAV‐BMP7 was recombined with the CMV promoter by Genechem Company. AAV with the GFP gene (AAV‐GFP) was used as a control. A stereotaxic instrument (RWD Life Science) was used for intramedullary injection. After the rats were anaesthetized and the T9 lamina was moved, a 34G Hamilton needle was stereotaxically inserted into the spinal cord (coordinates: 1 mm left or 1 mm right lateral from the central vessel, and 1.5 mm ventral from the dura). Both the left and the right lateral spinal cords were injected with 0.5 μl viral genome (1.04 × 10^13^ vector genomes/ml) at a rate of 0.5 μl/min using a micro‐syringe pump (RWD Life Science).

### 2.3 Spinal cord injury

Rats were anaesthetized with chloral hydrate (400 mg/kg, i.p.). After the lamina of T9 was removed, the spinal cords were exposed with the dura being intact. The animal was fixed on the modified Allen's impactor (RWD Life Science), with the T8 and T11 spinous processes clamped to stabilize the spine; a 10g rod was dropped at the exposed T9 spinal cord from a height of 5 cm. After the injury, animals with tail spasms and paralysis of both hindlimbs were ascertained successful modelling. Animals in the sham group underwent a T9 laminectomy without SCI. Animals in the SCI + AAV‐GFP and SCI + AAV‐BMP7 groups received AAV injection immediately before the injury. After a topical antibiotic was used, the incision was sutured layer by layer. Auxiliary urination was applied to the injured rats three times a day until the urinary retention disappeared. The animals were allowed to survive for 1 day or 1, 2, 3 or 4 weeks after the operation.

### 2.4 Quantitative real‐time PCR

Animals (*n* = 3 per group) were sacrificed at the indicated time points, and 0.5‐cm spinal cord (with the epicentre as a midpoint for injured rats) was collected for PCR analysis. Total RNA of the spinal cord was extracted by TRIzol Reagent (QIAGEN Science, catalog: 79306) and reverse‐transcribed into cDNA with a cDNA Synthesis Kit (Thermo Fisher Scientific, catalog: 00727497). RT‐qPCR was performed by a PCR System (Bio‐Rad Laboratories). Fold changes were calculated by the 2^−ΔΔCt^ method and normalized to β‐actin of the same sample. Data are presented as the mean ± SD from three repeats of each sample. The primer sequences were as follows: β‐actin forward, 5′‐TACCCAGGCATTGCTGACAG‐3′, reverse, 5′‐ACTCCTGCTTGCTGATCCAC‐3′; BMP7 forward, 5′‐GCCTGGACAACGAGGTG‐3′, reverse, 5′‐AGCCCAAGATGGACAGGA‐3′; CNPase forward, 5′‐TCCGAGGAGTACAAGCGTCT‐3′, reverse, 5′‐ACAGCTGCCATTGGTTCTTC‐3′; MBP forward, 5′‐GGCATCACAGAAGAGACCCTCAC‐3′, reverse, 5′‐GCCCGATGGAGTCAAGGATG‐3′.

### 2.5 Western blotting

Animals (*n* = 3 per group) were sacrificed, and 0.5‐cm spinal cord (with the epicentre as a midpoint for injured rats) was collected for total protein extraction. Proteins were separated by 8%–15% (w/v) SDS‐PAGE and transferred to polyvinylidene fluoride (PVDF) membranes. After blocking with 5% (w/v) non‐fat dried milk or 1% (w/v) gelatin, the membranes were incubated overnight at 4°C with the following primary antibodies: anti‐CNPase (1:2000, Abcam, catalog: ab6319), anti‐MBP (1:6000, Abcam, catalog: ab218011), anti‐BMP7 (1:500, Bioss, catalog: bs2242R), anti‐NF200 (1:1000; Abways, catalog: CY6657), anti‐STAT3 (1:; Cell Signaling, catalog: 30835s), anti‐p‐STAT3 (1:1000; Cell Signaling, catalog: 9145s), anti‐Smad1/5/9 (1:1000; Affinity, catalog: AF0614) and anti‐p‐Smad1/5/9 (1:1000; Cell Signaling, catalog: 13820). The next day, membranes were incubated with appropriate horseradish peroxidase (HRP)‐conjugated antibodies. After reacting with the chemiluminescent HRP substrate (Millipore), the immunoreactivity bands were photographed and analysed by a gel imaging system (Clinx Science Instrument). Each membrane was exposed five times continuously, from weak to strong, and then, suitable photographs were selected to avoid overexposure. Data are presented as the mean ± SD from three repeats of each sample.

### 2.6 Immunofluorescence

Rats (*n* = 3 per group) were deeply anaesthetized with chloral hydrate (400 mg/kg, i.p.) before perfusion with 4% paraformaldehyde. 0.5‐cm spinal cord (with the epicentre as a midpoint for injured rats) was collected and cut into 10‐µm sections transversely from rostral to caudal by a freezing microtome (Leica). 100 consecutive sections were collected as a group, with 1‐mm intervals between groups. Sections in the 3rd group were regarded as the injury epicentre. The sections were first washed in 0.3% Triton X‐100 for 15 min and then blocked in 5% bovine serum albumin (BSA) before incubation with the following primary antibodies: anti‐mouse APC (1:500, Abcam, catalog: ab16794), anti‐NF200 (1:200, Abways, catalog: CY6657), anti‐rabbit p‐STAT3 (1:200, CST, catalog: 9145s) and anti‐rabbit p‐Smad1/5/9 (1:200, CST, catalog: 13820). Subsequently, the sections were incubated with appropriate secondary antibodies (Cy5‐conjugated goat anti‐rabbit IgG [1:200, Servicebio, catalog: GB27303] and Cy3‐conjugated goat anti‐mouse IgG [1:200, Servicebio, catalog: GB21301]) diluted in PBS for 1 h at room temperature. Lastly, the stained sections were covered and photographed by fluorescence microscopy (OLYMPUS). The positive cells of NF200, APC, P‐smad 1/5/9 and P‐stat3 were counted automatically by using the IPP software. Five sections from the epicentre of each sample and five randomly selected white matter regions in each section were analysed.

### 2.7 Immunohistochemistry

Frozen sections from the epicentre were harvested as described in the Immunofluorescence subsection. Sections were washed in hydrogen peroxide (3%) before incubation with BSA and primary antibody (anti‐APC: 1:1000, Abcam, catalog: ab16794) diluted in PBS. After washing, sections were incubated with the corresponding biotin‐labelled secondary antibody diluted in PBS for 1 h. Subsequently, the sections were washed and immersed in the newly prepared DAB solution. The stained sections were placed on slides and dehydrated gradually before mounting with neutral gum. The tissues were visualized and photographed under a microscope (OLYMPUS). The positive cells of APC were counted automatically by using the IPP software. Five sections from the epicentre of each sample and five randomly selected white matter regions in each section were analysed.

### 2.8 G‐ratio analysis of myelin

Rats (*n* = 3 per group) were deeply anaesthetized, and 1‐mm^3^ fresh tissue from the injury epicentre was harvested within 3 min and put into TEM fixative (Servicebio) at 4°C for preservation and transportation. Then, samples were postfixed to avoid light with 1% OsO_4_ in 0.1 M PB (pH 7.4) for 2 h at room temperature, followed by dehydrating gradually in ethanol at room temperature. After embedding with EMBed 812 (SPI, catalog: 90529‐77‐4), samples were kept at 37°C overnight and moved to 65°C to polymerize for at least 48 h before cutting into 60‐ to 80‐nm sections with the ultramicrotome. Uranium acetate saturated alcohol solution (2%) and lead citrate (2.6%) were used to stain in sequence. Finally, samples were observed and images were captured under a TEM (HITACHI) at 1200× and 7000× G‐ratios of myelinated axons were measured with ImageJ. Five sections from the epicentre of each sample, and five white matter regions randomly selected from the ventral funiculus in each section were analysed.

### 2.9 BBB scores

The Basso‐Beattie‐Bresnahan (BBB) rating scale^21^ is a sensitive and reliable scale to assess the behavioural outcome of injured rats. The rats were placed in an open‐field environment and assessed from aspects of joint movement, coordination and physical stability. The test was performed on day 1 and day 7 after the operation and then weekly for 4 weeks by two trained researchers who were blind to the treatment group.

### 2.10 Statistical analysis

The unpaired Student's *t*‐test and ANOVA were used to analyse the significance of differences. Differences with *p* < 0.05 were regarded as statistically significant. All of the data are presented as the mean ± SD. The analyses were conducted and diagrams were constructed by GraphPad Prism 7.0 software.

## 3 RESULTS

### 3.1 SCI induced progressive demyelination with a reduction of BMP7

**FIGURE 1:**
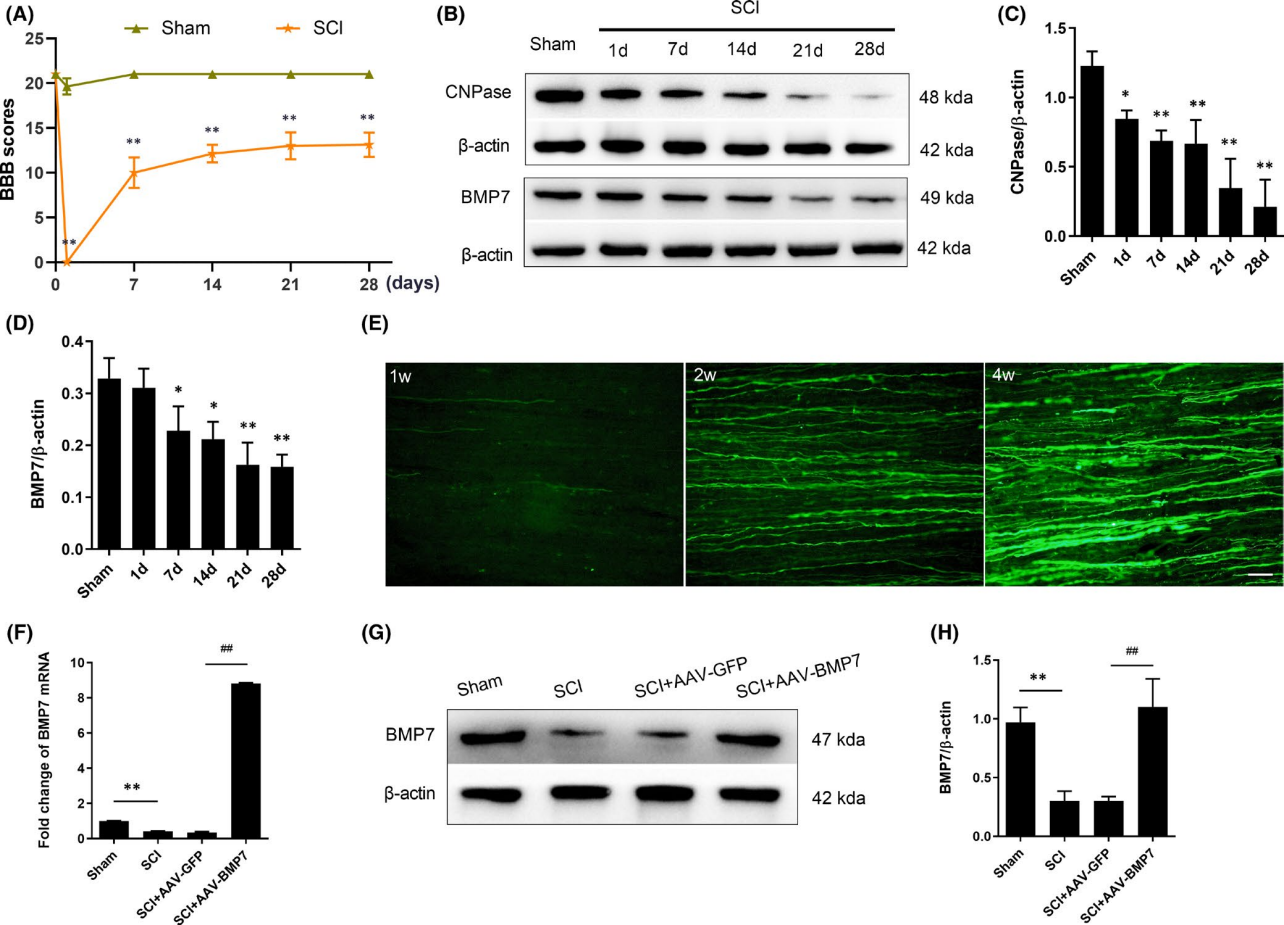
SCI caused CNPase loss and motor dysfunction with a reduction of BMP7; AAV‐BMP7 upregulated the expression of BMP7. (A) BBB scores of rats in the SCI group and the sham group at indicated time point. *n* = 8 per group. **p < 0.01 versus sham, unpaired Student's *t*‐test with Welch's correction. (B–D) Western blot results of CNPase and BMP7 from spinal cords of the sham group and the SCI group, and the quantification of bands normalized to β‐actin. *n* = 3 per group. *p < 0.05, **p < 0.01 versus sham, one‐way ANOVA. (E) Representative fluorescence images of GFP in the white matter of the injection site 1, 2 and 4 weeks after injection. Scale bar = 50 µm. (F) Fold change of BMP7 mRNA levels in each group at day 28 after the operation. *n* = 3 per group. **p < 0.01 vs sham, ^##^ *p* < 0.01 vs SCI + AAV‐GFP, one‐way ANOVA. (G, H) Representative Western blot results of BMP7 from the sham, SCI, SCI + AAV‐GFP and SCI + AAV‐BMP7 groups at day 28 after the operation and the quantification of bands normalized to β‐actin. *n* = 3 per group. **p < 0.01 vs sham, ^##^ *p* < 0.01 vs SCI + AAV‐GFP, one‐way ANOVA

The injured rats showed complete paralysis of hind limbs with a significant decrease in BBB scores on the first day after SCI (*p* < 0.01), indicating the success of SCI model. The injured rats stayed disabled, though partly recovered, for 28 days after SCI (*p* < 0.01). The self‐recovery occurred mainly during the first week after SCI and the BBB scores rose slightly in the following 3 weeks (Figure 1A). Although the neural function was recovered to a certain degree after SCI, the level of CNPase, which is a marker of OLs, reduced from the first day after SCI (*p* < 0.05) and continued to reduce until week 4 (Figure 1B,C). This means demyelination was progressive over a long period. Moreover, we found that BMP7 showed the same expression pattern as CNPase (Figure 1B,D) with a clear reduction over time. These observations motivated us to further explore whether the overexpression of BMP7 can affect OLs after SCI.

### 3.2 BMP7 was upregulated after the injection of AAV‐BMP7

To investigate the transfection duration of AAV9, we sacrificed the AAV‐GFP‐injected rats at days 7, 14 and 28 after the injection. Fluorescence images in the white matter indicated GFP in this area increased over time and reached its maximum at day 28 after the injection (Figure 1E). Besides, to confirm the overexpression of BMP7 in the injured rats, we measured the expression of BMP7 at the transcription (Figure 1F) and translation levels (Figure 1G,H). Compared with the control vector group, mRNA (*p* < 0.01) and protein (*p* < 0.05) levels of BMP7 were both significantly upregulated by AAV‐BMP7.

### 3.3 AAV‐BMP7 reduced the loss of OLs after SCI

**FIGURE 2:**
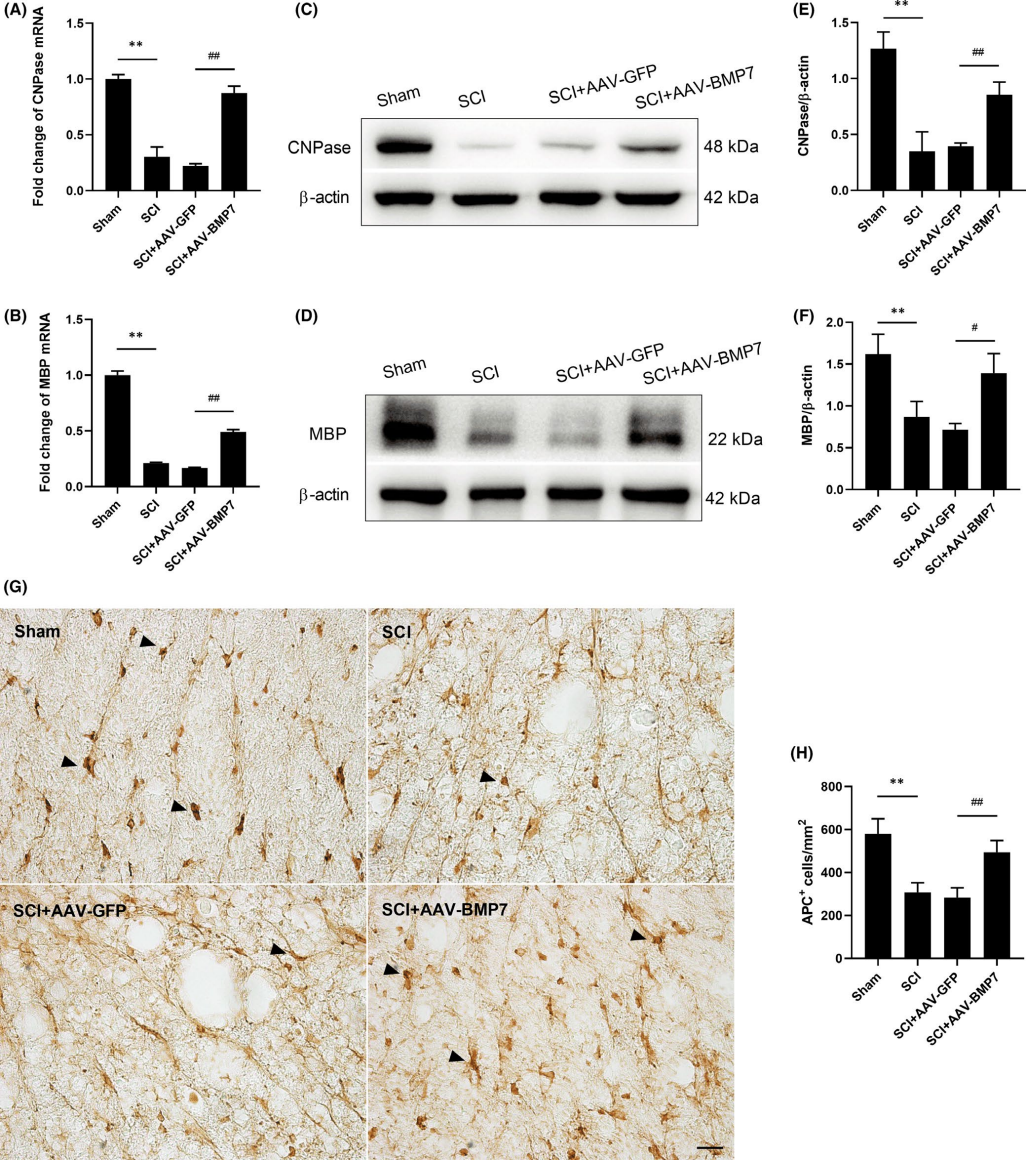
AAV‐BMP7 reduced the loss of OLs after SCI. (A, B) Fold change of CNPase and MBP mRNA levels of spinal cords from each group at day 28 after the operation. *n* = 3 per group. **p < 0.01 vs sham, ^##^ *p* < 0.01 vs SCI + AAV‐GFP, one‐way ANOVA. (C–F) Representative immunoblots of CNPase and MBP from the sham, SCI, SCI + AAV‐GFP and SCI + AAV‐BMP7 groups at day 28 after the operation and the quantification of bands normalized to β‐actin. *n* = 3 per group. **p < 0.01 vs sham, ^#^ *p* < 0.05, ^##^ *p* < 0.01 vs SCI + AAV‐GFP, one‐way ANOVA. (G, H) Representative immunohistochemical images of APC (arrows) in the ventral funiculus of the epicentre at day 28 after the operation. *n* = 3 per group. Scale bar = 20 µm. (H) The density of APC^+^ cells which was calculated from (G). *n* = 3 per group. ^##^ *p* < 0.01 vs SCI + AAV‐GFP, one‐way ANOVA

We then tested the expression pattern of CNPase, MBP and APC in each group at day 28 after the injection of AAV‐BMP7. CNPase decreased significantly at day 28 after SCI, which was consistent with the results shown in Figure 1A. Excitingly, this phenomenon was reversed to a certain degree when AAV‐BMP7 was applied, as mRNA and protein levels of both CNPase and MBP were increased (Figure 2A–F). Histological images of APC, a marker of OLs, also demonstrated an increasing trend in the SCI + AAV‐BMP7 group (Figure 2G,H).

### 3.4 AAV‐BMP7 alleviated demyelination after SCI

**FIGURE 3:**
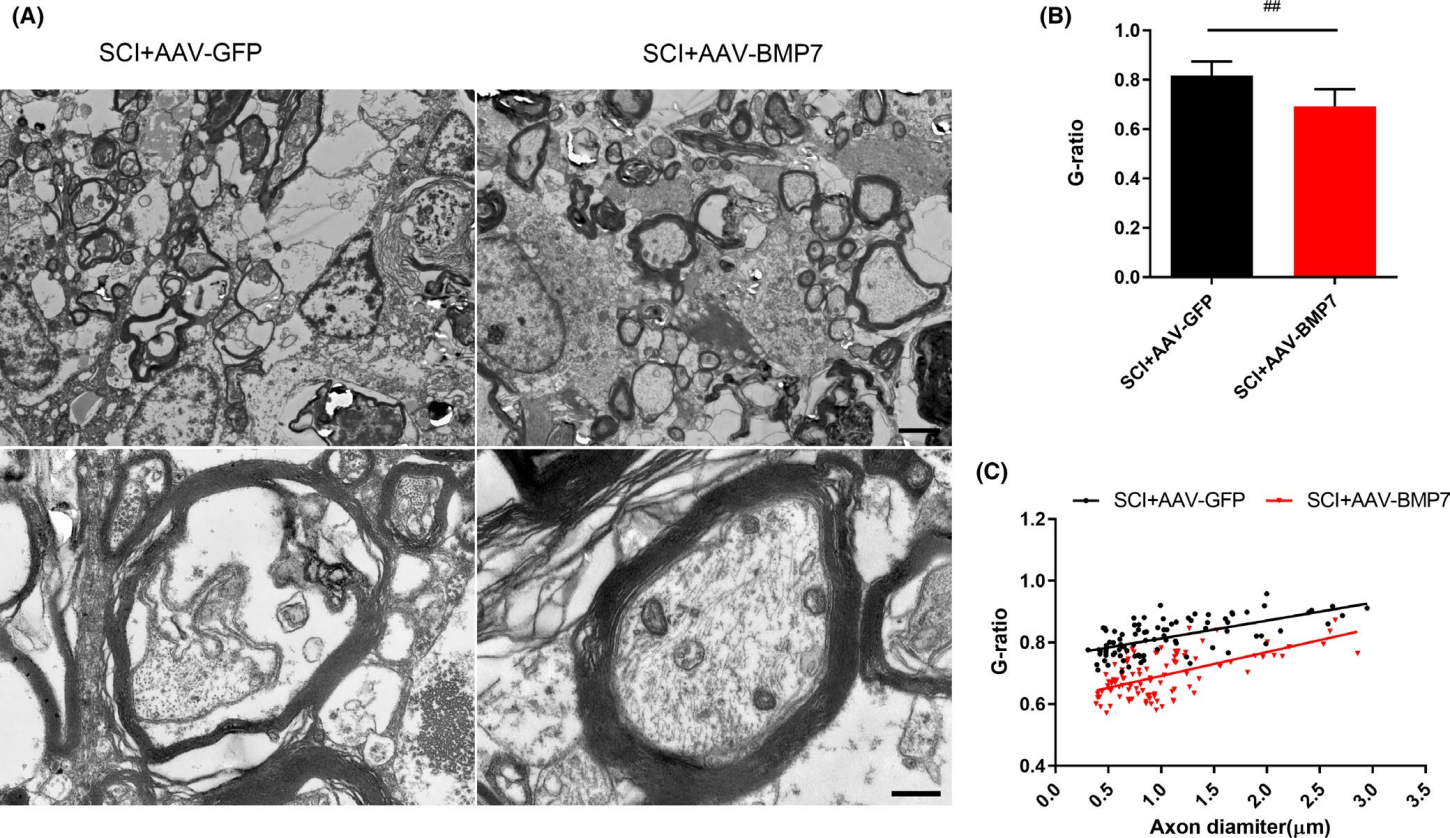
AAV‐BMP7 alleviated demyelination induced by SCI. (A) Representative TEM images in the ventral funiculus of epicentres from rats in the SCI + AAV‐GFP and SCI + AAV‐BMP7 groups, showing the myelin profiles at low power (1200×, the upper row) and high power (7000×, the lower row). Low power: scale bar = 0.5 µm; high power: scale bar = 2.5 µm. (B, C) G‐ratios of myelin with different diameters which were calculated from (A) *n* = 3 per group. ^##^ *p* < 0.01 vs SCI + AAV‐GFP, unpaired Student's *t*‐test

For further identification of the protective effects of AAV‐BMP7 on OLs, we observed the ultrastructure of myelin, which was formed by OLs, in the injured spinal cords. There were cavities in the white matter of both groups due to the damage. In the AAV‐GFP‐injected group, some of the myelin sheaths were thin, loose, irregular and even fractured, indicating they were degenerated. While in the SCI + AAV‐BMP7 group, myelin sheaths were thicker and denser, with fewer demyelinated axons (Figure 3A). G‐ratio, the ratio of the axonal diameter to the total outer diameter of nerve fibres, revealed that myelin in the AAV‐BMP7 group was thicker and more proportional to the axon diameters (Figure 3B,C; *p* < 0.01).

### 3.5 AAV‐BMP7‐activated Smad1/5/9 and STAT3 in OLs

**FIGURE 4:**
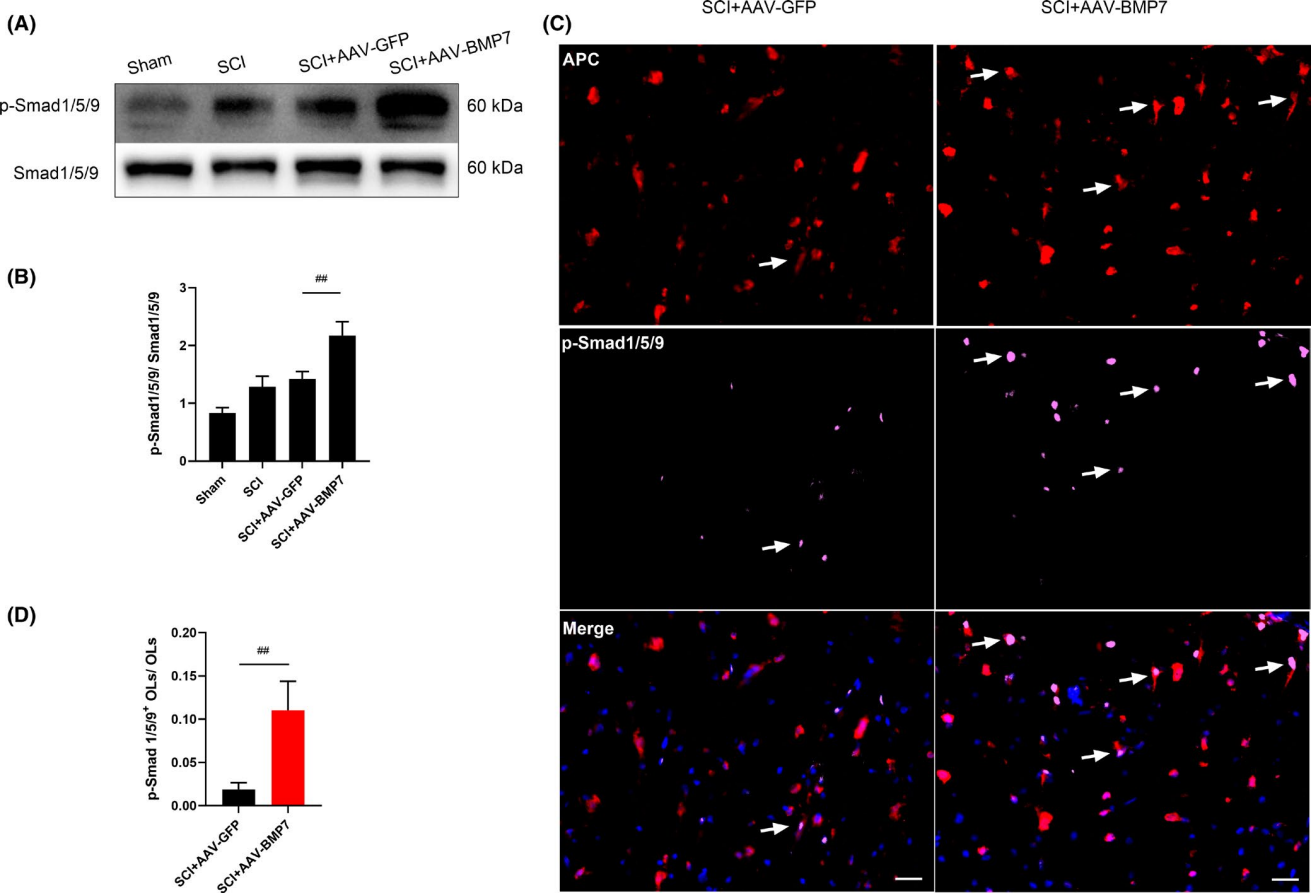
AAV‐BMP7‐activated Smad1/5/9 in OLs. (A, B) Representative immunoblots of p‐Smad1/5/9 and Smad1/5/9 from the sham, SCI, SCI + AAV‐GFP and SCI + AAV‐BMP7 groups at day 28 after the operation and the quantification of bands normalized to β‐actin. *n* = 3 per group. ^##^ *p* < 0.01 vs SCI + AAV‐GFP, one‐way ANOVA. (C) Representative fluorescence micrographs of APC (red)/p‐Smad1/5/9 (pink) double‐staining (arrows) in the ventral funiculus of the epicentre at day 28 after SCI. Scale bar = 20 µm. (D) The ratio of p‐Smad1/5/9^+^ OLs to OLs (marked by APC) which was calculated from (C) *n* = 3 per group. ^##^ *p* < 0.01 vs SCI + AAV‐GFP, unpaired Student's *t*‐test

**FIGURE 5:**
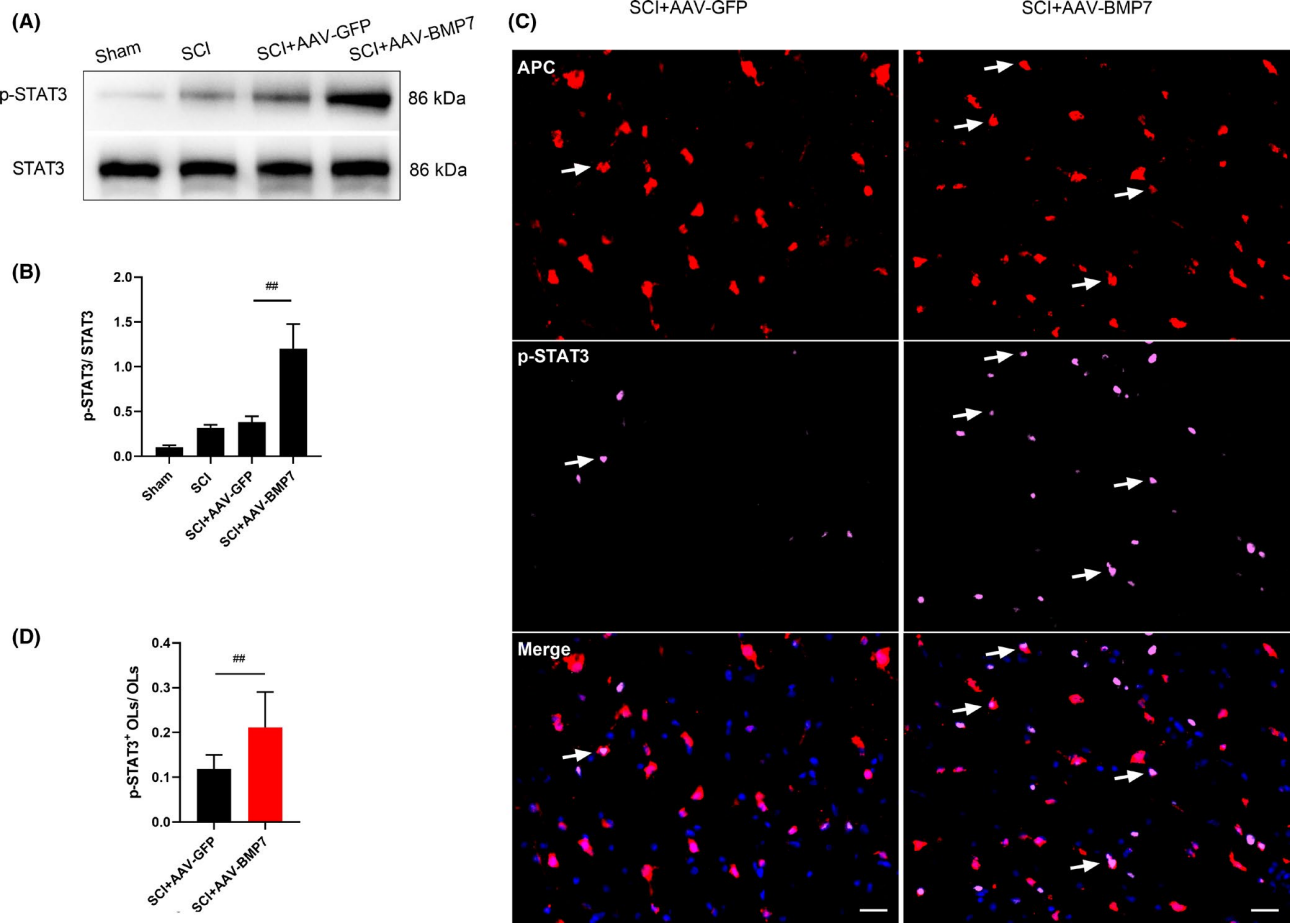
AAV‐BMP7‐activated STAT3 in OLs. (A, B) Representative immunoblots of p‐STAT3 and STAT3 from the sham, SCI, SCI + AAV‐GFP and SCI + AAV‐BMP7 groups at day 28 after the operation and the quantification of bands normalized to β‐actin. *n* = 3 per group. ^##^ *p* < 0.01 vs SCI + AAV‐GFP, one‐way ANOVA. (C) Representative fluorescence micrographs of APC (red)/p‐STAT3 (pink) double‐staining (arrows) in the ventral funiculus of the epicentre at day 28 after SCI. Scale bar = 20 µm. (D) The ratio of p‐STAT3^+^ OLs and OLs (marked by APC) which was calculated from c. *n* = 3 per group. ^##^ *p* < 0.01 vs SCI + AAV‐GFP, unpaired Student's *t*‐test

To further explore the mechanism of how AAV‐BMP7 protects OLs after SCI, the levels of two classical downstream signals of BMPs, Smad1/5/9 and STAT3, were analysed. The relative amounts of both p‐Smad1/5/9 (Figure 4A,B) and p‐STAT3 (Figure 5A,B) in AAV‐BMP7‐injected animals were significantly higher than those in the AAV‐GFP injected ones (*p* < 0.01).

While there were occasionally some cells simultaneously marked by p‐Smad1/5/9 and APC in the SCI + AAV‐GFP group, more p‐Smad1/5/9 was induced and co‐located with APC in the AAV‐BMP7‐injected group (Figure 4C). The proportion of p‐Smad1/5/9^+^ OLs in the AAV‐BMP7 group was much higher than that in the AAV‐GFP group (Figure 4D; *p* < 0.01). Interestingly, p‐STAT3 showed the same trend in OLs. AAV‐BMP7 induced more STAT3 phosphorylated in OLs (Figure 5C,D). These results demonstrate that p‐Smad1/5/9 and p‐STAT3 may have a role in the protective effects of BMP7 on OLs.

### 3.6 AAV‐BMP7 protected axons and improved motor function after SCI

**FIGURE 6:**
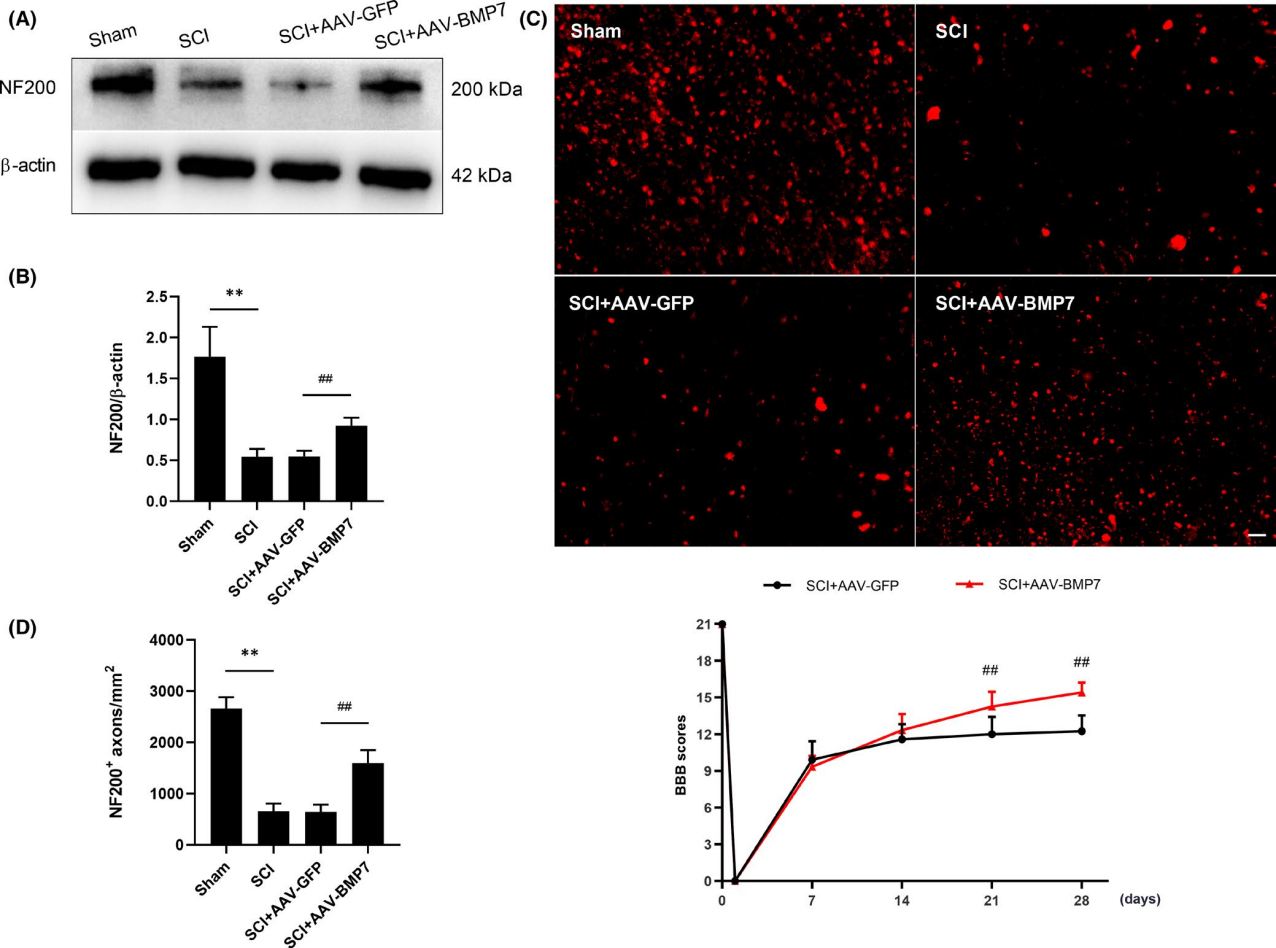
AAV‐BMP7‐injected rats showed more NF200^+^ axons and better recovery of locomotor function after SCI. (A, B) Representative immunoblots of NF200 from the sham, SCI, SCI + AAV‐GFP and SCI + AAV‐BMP7 groups at day 28 after the operation and the quantification of bands normalized to β‐actin. *n* = 3 per group. **p < 0.01 vs sham, ^##^ *p* < 0.01 vs SCI + AAV‐GFP, one‐way ANOVA. (C) Representative fluorescence micrographs of NF200 in the ventral funiculus of the epicentre at day 28 after SCI. Scale bar = 20 µm. (D) The density of NF200^+^ axons was calculated from c. *n* = 3 per group. **p < 0.01 vs sham, ^##^ *p* < 0.01 vs SCI + AAV‐GFP, one‐way ANOVA. (E) BBB scores of rats in the SCI + AAV‐GFP group and the SCI + AAV‐BMP7 group at indicated time points. *n*=12 per group. ^##^ *p* < 0.01 vs SCI + AAV‐GFP, unpaired Student's *t*‐test with Welch's correction

The number and density of NF200‐positive axons were significantly reduced at day 28 after SCI, but increased when AAV‐BMP7 was applied (Figure 6A–D). NF200 immunostaining revealed that axons were sparse, irregular and swollen in the SCI and SCI + AAV‐GFP groups, while those in the SCI + AAV‐BMP7 group were numerous, orderly and uniformly distributed (Figure 6C,D). Consistent with the results in Figure 1, SCI rats suffered from neurological dysfunction and later partly recovered. There was no significant difference in the recovery of all injured rats in the first 2 weeks. The injection of AAV‐BMP7 promoted functional recovery in the latter phase of SCI, and the BBB scores of AAV‐BMP7‐injected animals reached 14.25 and 15.42 at day 21 and day 28, respectively, after SCI (both *p* < 0.01), which were significantly higher than those of AAV‐GFP‐injected animals (12 and 12.25, respectively; Figure 6E).

## 4 DISCUSSION

The present study showed that BMP7 can partly reverse OLs loss, reduce demyelination, protect axons and improve motor function after SCI. Although it has been evidenced that BMP7 can protect Schwann cells and cultured OLs in previous studies, we found for the first time that BMP7 can protect OLs and myelin against damage from SCI in vivo.

The main role of OLs is to form myelin, which wraps around axons and is essential for the saltatory conduction of action potentials and the normal function of neurons.^22^, ^23^ Researchers have elucidated that apoptosis of OLs continues during an extended period after SCI, resulting in chronic demyelination.^24^, ^25^ The loss of myelin exposes axons, impairs their conductive capacity and ultimately causes deficits in movement, motor coordination, balance and cognition.^26^, ^27^, ^28^ Similarly, in the present study, the levels of CNPase, a marker of OLs, decreased until 4 weeks after SCI, indicating OLs suffered from chronic damage. Although partially recovered, animals in our study stayed disabled for more than 4 weeks after SCI, which might be attributed partly to the chronic demyelination.

A growing number of studies have suggested BMP7 exerts neuroprotective effects on the nervous system. BMP7 has been reported to protect neurons,^10^, ^11^, ^12^, ^13^ promote the differentiation of OLs,^17^ inhibit apoptosis of cultured OLs^19^ and reduce the loss of Schwann cells, which is essential for peripheral myelination.^20^ However, the expression and function of BMP7 in the nervous system differed in some other studies. BMP7 has been shown to alternate the fate of neuroepithelial cells from neurogenesis to astrocytogenesis.^29^ Some studies have demonstrated that SCI induces the expression of BMP7.^30^, ^31^ Another study has shown that BMP7 increases rapidly in the demyelinating spinal cord lesions, thus activating astrocytes, promoting glial scar formation and increasing the expression of CSPGs, which may inhibit remyelination.^32^ In the peripheral nervous system, researchers have found that BMP7 negatively regulates peripheral myelin gene expression by activating p38MAPK in Schwann cells.^33^ In the present study, we found BMP7 was decreased after SCI, and AAV‐BMP7 partly reversed OLs loss, alleviated demyelination, protected axons and promoted functional recovery. Although OLs are essential to maintain the normal function of the nervous system, more direct results are required to conclude that the increase of OLs is the main factor for the increase of axons and functional recovery in this study. Other cells such as neurons and astrocytes may also be affected by BMP7 and contribute to the neurological functional recovery.

A previous study has demonstrated that acute delivery of BMP7 significantly improves neurological deficits of IR rats.^11^ Rats that received a single dose of intravenous BMP7 24 h after stroke showed a decrease in body asymmetry and an increase in locomotor activity.^34^ SCI rats that administrated BMP7 for 3 days showed an increase in neuronal sparing in the ventral horn of the spinal cord.^10^ In our present study, we applied adeno‐associated virus (AAV) as a vector to obtain stable and sustained expression of BMP7. Significantly, overexpression of BMP7 occurred 2 weeks after SCI and the results showed AAV‐BMP7 promoted functional recovery in the latter phase of SCI. It is reasonable to speculate that if applied earlier, AAV‐BMP7 may show better protection in the acute phase.

Surprisingly, a body of studies have revealed that different subtypes of BMPs exert different effects on OLs, even though they share the same receptors. BMP4, which may be the best‐studied subtype of BMPs, was consistently reported to obstruct the differentiation and maturation of OLs.^35^ In vitro, oligodendrocyte precursor cells (OPCs) cultured with BMP4 could only differentiate into immature OL phenotype with no myelin proteins.^36^ Application of BMP4 promoted cultured neural stem cells (NSCs) to differentiate into astrocytes, while inhibiting the production of OLs.^30^ In the transgenic mice that overexpressed BMP4, the density of OLs was decreased remarkably in the CNS.^37^ The mechanisms that underlie these paradoxical effects of BMPs remain unclear. BMPRIa and BMPRIb are BMP receptors that mediate different opposing responses even within the same cells in the developing nervous system^38^, ^39^, ^40^, ^41^ and the injured spinal cord.^42^ Since BMPRIa and BMPRIb have been reported to exist in OLs and OPCs,^43^, ^44^ we speculate that the contradictory effects of BMP4 and BMP7 on OLs may depend on the competition between the two receptors. However, this hypothesis requires verification.

Smad1/5/9 is considered pivotal intracellular effectors of BMPs.^45^, ^46^ Previous studies have found that BMP7 inhibits fibrosis by phosphorylating Smad1/5/9 in different diseases.^47^, ^48^, ^49^ Interestingly, some researchers have proved that the knockdown of smad1/5/9 contributes to abnormal neurodevelopment.^50^ In adult rats, BMP7/Smad1/5/9 signalling promoted axon regeneration after SCI.^51^ Moreover, a recent study has indicated that BMP7 can suppress excessive scar formation by activating Smad1/5/9.^52^ In the present study, Smad1/5/9 was activated after AAV‐BMP7 injection, and more p‐Smad1/5/9 was observed in OLs, suggesting that Smad1/5/9 might be involved in the protection of BMP7 on OLs and myelin.

STAT3, which is strongly linked to cell proliferation, differentiation, inflammation and survival, is extensively involved in the CNS.^53^ Previous studies have found that CNTF and PDGF can enhance OL progenitor survival by initiating phosphorylation of STAT3.^54^ A recent study has revealed that p‐STAT3 induced by cytokines promotes the differentiation of OPCs into mature OLs and increases the survival of OLs.^55^, ^56^ NG2 cell that can differentiate into OLs has been reported to express p‐STAT3, and the peak p‐STAT3 expression is synchronized with the strongest NG2 cell proliferation,^57^ indicating that this signalling pathway may promote the differentiation of OLs. In addition, BMPs has been reported to activate STAT to regulate glial differentiation.^58^ Similarly, in the present study, overexpression of BMP7 induced more p‐STAT3 in OLs and resulted in beneficial effects on OLs and locomotor function.

Both p‐STAT3 and p‐Smad1/5/9 were activated after SCI, while BMP7 was reduced. We suspect that these signallings may be affected by other BMPs simultaneously. In the light of our findings that p‐STAT3 and p‐Smad1/5/9 in OLs are upregulated by AAV‐BMP7 post‐SCI, we hypothesize that both Smad1/5/9 and STAT3 may be responsible for the protection of BMP7 on OLs after SCI.

In summary, our results showed that in SCI rats, injection of AAV‐BMP7 increased the number of OLs, reduced demyelination, protected axons and was accompanied with higher BBB scores. Moreover, both Smad1/5/9 and STAT3 were activated in OLs, and they were probably partly responsible for the beneficial effects of BMP7 on OLs and functional recovery. However, it remains unclear whether the promotion of OLs differentiation or inhibition of OLs apoptosis is the reason for the increase of OLs in this experiment and further studies are required for verification. Our results indicate that BMP7 may serve as a new therapeutic target for SCI.

## CONFLICT OF INTEREST

The authors have no conflict of interest to declare.

## AUTHOR CONTRIBUTIONS

**Shuxin Liu:** Conceptualization (equal); Formal analysis (equal); Writing‐original draft (lead). **Wei Zhang:** Formal analysis (equal); Methodology (equal); Writing‐review & editing (equal). **Lin Yang:** Methodology (equal); Writing‐review & editing (equal). **Fan Zhou:** Funding acquisition (equal); Writing‐review & editing (equal). **Peng Liu:** Formal analysis (equal); Methodology (equal). **Yaping Wang:** Conceptualization (equal); Funding acquisition (equal); Writing‐review & editing (equal).

## Data Availability

The data and materials that support the findings of this study are available from the corresponding author, Yaping Wang, upon reasonable request.
